# Amphipathic Phenylalanine-Induced Nucleophilic–Hydrophobic Interface Toward Highly Reversible Zn Anode

**DOI:** 10.1007/s40820-024-01380-x

**Published:** 2024-03-28

**Authors:** Anbin Zhou, Huirong Wang, Fengling Zhang, Xin Hu, Zhihang Song, Yi Chen, Yongxin Huang, Yanhua Cui, Yixiu Cui, Li Li, Feng Wu, Renjie Chen

**Affiliations:** 1 Beijing Key Laboratory of Environmental Science and Engineering, School of Materials Science and Engineering, Beijing Institute of Technology, Beijing, 100081 People’s Republic of China; 2 Advanced Technology Research Institute, Beijing Institute of Technology, Jinan, 250300 People’s Republic of China; 3 Collaborative Innovation Center of Electric Vehicles in Beijing, Beijing, 100081 People’s Republic of China; 4 Institute of Electronic Engineering, China Academy of Engineering Physics, Mianyang, 621900 People’s Republic of China

**Keywords:** Zn anode, Phenylalanine, Adsorption energy, Solvation sheath

## Abstract

Aqueous Zn^2+^-ion batteries (AZIBs), recognized for their high security, reliability, and cost efficiency, have garnered considerable attention. However, the prevalent issues of dendrite growth and parasitic reactions at the Zn electrode interface significantly impede their practical application. In this study, we introduced a ubiquitous biomolecule of phenylalanine (Phe) into the electrolyte as a multifunctional additive to improve the reversibility of the Zn anode. Leveraging its exceptional nucleophilic characteristics, Phe molecules tend to coordinate with Zn^2+^ ions for optimizing the solvation environment. Simultaneously, the distinctive lipophilicity of aromatic amino acids empowers Phe with a higher adsorption energy, enabling the construction of a multifunctional protective interphase. The hydrophobic benzene ring ligands act as cleaners for repelling H_2_O molecules, while the hydrophilic hydroxyl and carboxyl groups attract Zn^2+^ ions for homogenizing Zn^2+^ flux. Moreover, the preferential reduction of Phe molecules prior to H_2_O facilitates the *in situ* formation of an organic–inorganic hybrid solid electrolyte interphase, enhancing the interfacial stability of the Zn anode. Consequently, Zn||Zn cells display improved reversibility, achieving an extended cycle life of 5250 h. Additionally, Zn||LMO full cells exhibit enhanced cyclability of retaining 77.3% capacity after 300 cycles, demonstrating substantial potential in advancing the commercialization of AZIBs.

**Supplementary Information:**

The online version contains supplementary material available at 10.1007/s40820-024-01380-x.

## Highlights

- The amphipathic phenylalanine-adsorbed layer contributes to form a nucleophilic–hydrophobic interface that homogenizes Zn^2+^ flux while repelling H_2_O molecules from contacting Zn anode.
- The preferential reduction of phenylalanine (Phe) prior to H_2_O facilitates *in situ* formation of an organic–inorganic hybrid solid electrolyte interphase, enhancing the interfacial stability.
- Benefiting from the triple protection of Phe, the Zn||Zn and Zn||LMO cells display significantly improved electrochemical performances, even at extreme diluted electrolytes.

**Supplementary Information:**

The online version contains supplementary material available at 10.1007/s40820-024-01380-x.

## Graphical abstract

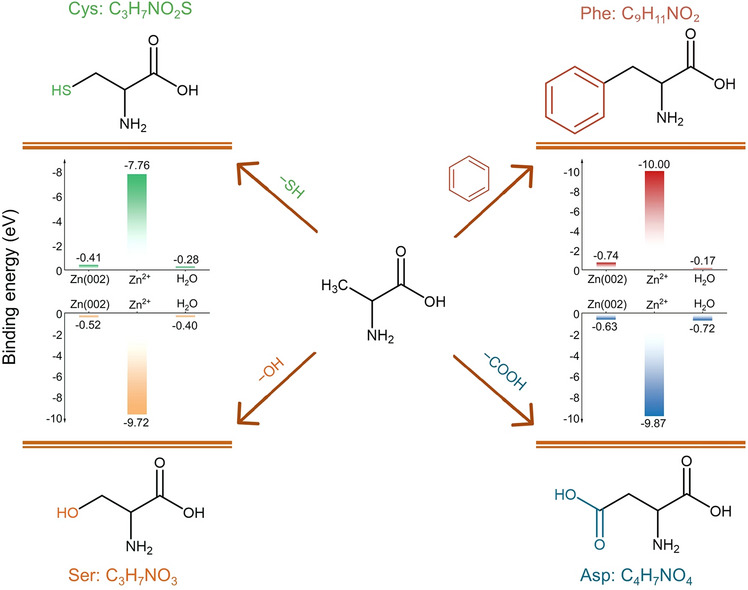

## Introduction

Wearable and implantable electronics have rapidly evolved to meet the growing demands of modernized health care [1–3], including devices like smartwatches, cochlear implants, and cardiac pacemakers [4]. The sustained operation of implanted medical devices heavily relies on safe rechargeable batteries powering electronic circuitry for data acquisition, processing, and transmission [5]. Notably, aqueous Zn^2+^-ion batteries (AZIBs) with inherently safe near-neutral electrolytes, superior biocompatibility, and higher energy density have gained significant attention [6–9]. However, the practical applications encounter some challenges such as dendrite growth and corrosion of Zn anodes, compromising long-term reliability [10–12]. Dendrite growth could puncture the separator and leads to battery short circuits, while Zn anode corrosion consumes active Zn and electrolyte, shortening its service life [13–15].

To enhance Zn anode reliability by inhibiting parasitic reactions or guiding uniform Zn deposition, various strategies, including electrode structure design [16–19], electrode interface modification [20–22], and electrolyte optimization [23–26], have been explored. Among these, the introduction of functional additives stands out as a feasible and effective approach with a substantial scale effect [27–29]. Previously, multiple mechanisms involving functional additives have demonstrated their role in achieving dendrite-free Zn anodes. For instance, the introduction of polar solvents like N-methyl-2-pyrrolidone (NMP) [30], N, N-dimethylformamide (DMF) [31], and acetone (DMK) [32] effectively optimizes the Zn^2+^ solvation sheath by replacing coordinated H_2_O, thereby restraining H_2_O-induced parasitic reactions and enhancing Zn reversibility. Additionally, the introduction of positively charged molecules with electrostatic preferential adsorption into the electrolyte induces an emerging progressive nucleation mechanism [27, 33, 34]. This mechanism stabilizes the Zn anode by homogenizing electric fields and regulating Zn deposition behavior. However, a single regulation strategy targeting solvation structure or molecule adsorption may not fundamentally shield the Zn anode from corrosion caused by free H_2_O molecules [35].

Hence, it is crucial to explore multifunctional additives that simultaneously reduce H_2_O molecule reactivity while constructing a robust hydrophobic protective interface. Amino acids, ubiquitous biomolecules, have gained attention for their non-toxicity, biodegradability, and extensive use in the chemical industry as complexing agents and corrosion inhibitors [36, 37]. These molecules possess hydrophilic amino and carboxyl groups at either end and hydrophobic groups (e.g., methyl, isopropyl) in the middle, making them amphipathic compounds [38]. Their electron-rich nature enables excellent adsorption on metal electrode surfaces [37]. The hydrophilic units reduce interfacial free energy between the Zn electrode and electrolyte, facilitating Zn^2+^ transfer [39]. The hydrophobic groups form a local hydrophobic film of closer to the electrolyte, suppressing water-mediated parasitic side reactions [39]. Importantly, the precisely modifiable structure of hydrophobic groups allows for customizable additive design.

**Fig. 1:**
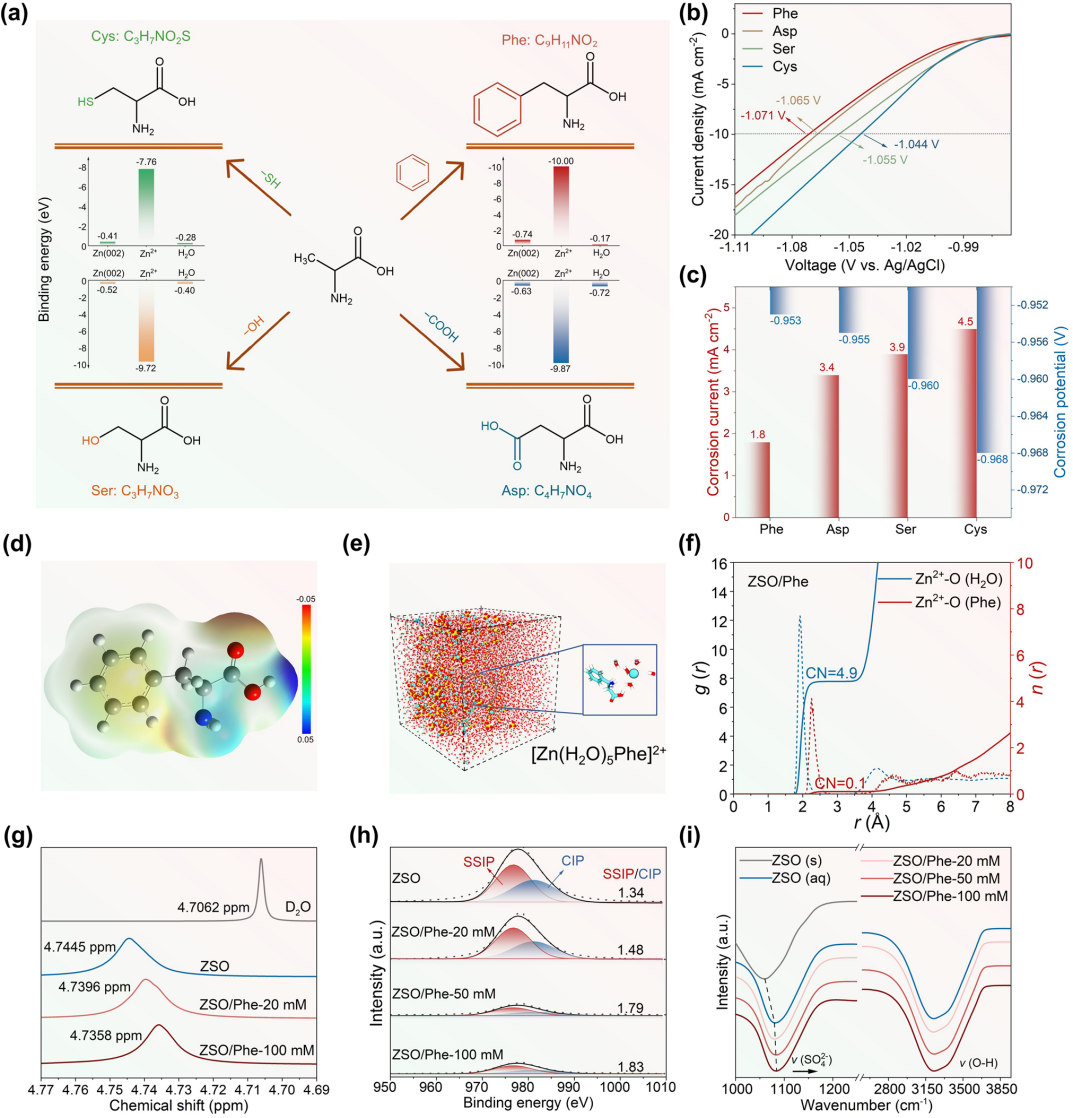
Characterization of electrolyte system. **a** Schematic illustration for various derivatives of amino acids. **b** LSV curves and **c** corrosion current density and potential derived from Tafel plots in 20 mmol L^−1^ Phe/Asp/Ser/Cys additives electrolytes. **d** Electrostatic potential mapping of Phe molecule. **e** Images of ZSO/Phe system obtained from molecular dynamics simulations. **f** Radial distribution functions for Zn^2+^-O (H_2_O) and Zn^2+^-O (Phe) in ZSO/Phe electrolyte. **g** ^2^H NMR spectra of H_2_O from pure D_2_O, ZSO, and ZSO/Phe system. **h** Raman spectra and **i** FTIR spectra for ZSO and ZSO/Phe system with various concentrations

Based on the representative alanine, various derivatives with unique nucleophilic and hydrophobic characteristics can be created by grafting benzene ring, carboxyl, hydroxyl, and sulfhydryl groups onto the methyl, such as phenylalanine (Phe), aspartic acid (Asp), serine (Ser), and L-cysteine (Cys). Recent studies have shown that the aromatic molecules, such as benzoic acid, 3-hydroxytyramine hydrochloride, isophthalaldehyde, and 1,3-benzenedisulfonic acid disodium salt, embrace higher adsorption energy and stronger chemisorption effect on the Zn surface that promotes an even electric field distribution at electrode/electrolyte interface, inducing the uniform Zn deposition [40]. Therefore, the Phe featuring benzene ring ligands was introduced into AZIBs as electrolyte additive. The theoretical simulation and experimental evidence demonstrate that Phe (Figs. 1a and S1-S3) exhibits superior nucleophilic ability to coordinate with Zn^2+^ ions, efficiently regulating solvation to weaken H_2_O molecule reactivity. Moreover, the unique lipophilicity of aromatic amino acids enables Phe to possess higher adsorption energy on the Zn (002) facet, contributing to form an nucleophilic–hydrophobic protective interphase [40]. The hydrophobic benzene ring ligands within Phe molecules form a hydrophobic film that repels H_2_O molecules. Simultaneously, the adjacent hydrophilic carboxyl and hydroxyl groups attract Zn^2+^ ions to migrate to the Zn anode surface, guiding uniform Zn nucleation along the favorable (002) plane. Consequently, the addition of Phe at a concentration of 20 mmol L^−1^ demonstrates a beneficial impact by reducing the onset of hydrogen evolution (Fig. 1b), decreasing the corrosion current density, and concurrently increasing the corrosion potential (Figs. 1c and S4), which surpasses that of other derivatives in terms of corrosion inhibition. Finally, Zn||Zn symmetrical cells assembled using Phe-modified electrolytes exhibit enhanced Zn anode reversibility, achieving an ultralong cycle life of 5250 h at 2 mA cm^−2^, 2 mAh cm^−2^, nearly 65 times higher than that in blank electrolytes, confirming its practical feasibility in AZIBs.

## Experimental and Calculation

### Preparation of Electrolytes and Electrodes

0.2 and 0.02 mol ZnSO_4_·7H_2_O (Aladdin, > 99.99%) were dissolved in 100 mL deionized water, Phe (Adamas, > 99%) solution (0.02 mol L^−1^) to obtain 2 mol L^−1^ ZnSO_4_ electrolyte, 0.2 mol L^−1^ ZnSO_4_-based dilute electrolyte, and 0.02 mol L^−1^ Phe containing electrolyte, respectively. 0.1 mol Li_2_SO_4_ (Macklin, > 99.9%) was dissolved in above solution to obtain hybrid electrolyte. Commercial Zn foil (100 µm) polished with sandpaper was cut into 11-mm-diameter disks for preparing Zn anode. LiMn_2_O_4_ powders (Shenzhen kejing Co., Ltd), super-P carbon black (Nanjing XFNANO Materials Tech Co., Ltd), and polyvinylidene fluoride were mixed to form slurry at a weight ratio of 8:1:1. Then the slurry was coated onto Ti foil at a mass loading of 2.5 mg cm^−2^ and dried at 60 °C for 12 h to obtain LMO cathode.

### Material Characterizations

Nuclear magnetic resonance (NMR, Bruker AVANCE III HD 600), Fourier transform infrared spectroscopy (FTIR, Bruker Alpha spectrometer), and Raman (Bruker RFS100/S) were conducted to characterize the electrolyte structure. The transmission electron microscopy (TEM, JEOL JEM 2800F), time-of-flight secondary ion mass spectrometer (TOF–SIMS, PHI nano TOF II), and X-ray photoelectron spectrometer (XPS, PHI-1600) with Ar^+^ etching were employed to analyzed the solid electrolyte interphase (SEI). The scanning electron microscopy (SEM, Hitachi SU-70), atomic force microscopy (AFM, Bruker Corp., Dimension Icon), and *in situ* optical microscope (Leica DVM6) were performed to investigate the Zn plating morphology. The *in situ* X-ray diffraction (XRD, Bruker AXS, WI, USA) and *in situ* differential electrochemical mass spectrometry (DEMS) were applied to characterize the phase structure change and monitor the H_2_ production, respectively, during charging/discharging process.

### Electrochemical Characterizations

CR2032-type coin cells (canrd, Canrd Technology Co. Ltd.) were assembled for Zn||Zn, Zn||Cu and Zn||LMO cells with glass fiber membrane soaked in 150 μL solution (DLAB Scientific Co., Ltd) to test the long-term galvanostatic cycling on a Neware battery test system (CT-4008 T-5V50mA-164, Shenzhen, China). Electrochemical impedance spectroscopy (EIS), cyclic voltammetry (CV) profiles, three-electrode-based measurements of Tafel plots, and linear sweep voltammetry (LSV) were performed on CorrTest CS350 electrochemical workstation (Wuhan Corrtest Instruments Corp., Ltd.).

### Molecular Dynamics Simulations

The molecular dynamics simulations were conducted through GROMACS [41] with AMBER force field [42]. The MD parameters for SO_4_^2−^ and amino acid molecule were generated through Sobtop [43], and the corresponding atom charges were based on RESP charges. The H_2_O molecule was simulated with the simple point charge model. The initial size of box was 10 × 10 × 10 nm^3^, and periodic boundary conditions were set in XYZ directions. The simulation cells contained 8200 H_2_O, 300 ZnSO_4_, and 3 Phe. The electrostatic interactions were computed using PME methods. A cutoff length of 1.0 nm was used in the calculation of electrostatic interactions and non-electrostatic interactions in real space. The integration time step was 1 fs. The system was annealed from 0 to 298 K over a period of 0.5 ns, followed by running for another 2.0 ns to reach equilibrium. The temperature and pressure coupling was performed with V-rescale and Berendsen method, respectively. Finally, a 10 ns production simulation was carried out for post-processing analysis. The pressure coupling method in production simulation period was changed to Parrinello–Rahman.

### Quantum Chemistry Calculations

Quantum chemistry calculations were conducted through Gaussian (G09) program. The structure optimization and energy calculations were performed at B3LYP-D3(BJ)/def2-SVP level. The calculations were carried out with the implicit universal solvation model based on solute electron density (SMD). The orbit was analyzed by Multiwfn package and VMD package [43].

### Density Functional Theory Calculations

First-principles calculations were performed using the Castep module in Materials Studio 2019 with density functional theory [44]. The Perdew–Bruke–Ernzerh of exchange–correlation functional of the generalized gradient approximation (GGA) was adopted, and the cutoff energy with the value of 800 eV was used in all the calculations. The Γ-centered k-point grids were used for Brillouin zone integrations. The convergence criterion for the electronic structure iteration was set at 1 × 10^−5^ eV, while the geometry optimization was set to be 0.02 eV Å^−1^ on force. The 7 × 7 supercell was used to model the Zn (002) surface, and a vacuum thickness of 15 Å was applied. The atoms in the top two layers were free to simulate surface state, and the atoms in the other layers were fixed during calculation.

## Results and Discussion

### Characterization of Electrolyte System

The highly electronegative carboxylate groups within the Phe molecule exhibit robust nucleophilic characteristics due to the regional electron enrichment induced by benzene ring ligands (Fig. 1d), allowing them effectively coordinate with Zn^2+^ ions and optimizing the Zn^2+^ solvation environment [45]. Subsequently, molecular dynamics simulations were conducted to explore the structure of the Zn^2+^ solvation sheath on bare ZnSO_4_ electrolyte (ZSO) and Phe-modified ZnSO_4_-based electrolyte (ZSO/Phe). As obtained from the radial distribution functions (RDFs) for Zn–O (H_2_O) in ZSO electrolyte (Figs. S5 and S6), a characteristic peak emerged at around 2 Å with an average coordination number (CN) reaching 5.2, suggesting that there are approximately six H_2_O molecules coordinating with Zn^2+^ within the primary Zn^2+^ solvation shell. In contrast, after introducing Phe additive into the electrolyte, the Phe molecule intrudes the primary Zn^2+^ solvation structure and forms chelation with Zn^2+^ ions, as evidenced by the electrolyte box image of ZSO/Phe electrolyte system (Fig. 1e). Additionally, the peaks of Zn–O originated from both Phe and H_2_O are positioned approximately 2 Å away from Zn^2+^, and the CN of Zn–O attributed to Phe and H_2_O show around 0.1 and 4.9, respectively (Fig. 1f). These results further validate the ability of Phe molecules to integrate into the primary Zn^2+^ solvation sheath and displace the partially solvated H_2_O molecules. Typically, solvated H_2_O molecules are deemed thermodynamically unstable at Zn deposition potential, representing a major contributing factor to the hydrogen evolution reaction (HER) [46]. Therefore, the orchestrated regulation of the Zn^2+^ solvation sheath is expected to curtail corrosion reactions induced by water molecules.

To delve deeper into the interaction mechanism of Phe additive in ZnSO_4_-based electrolyte, a series of analyses were conducted, including liquid-state NMR, FTIR, and Raman spectroscopy. As displayed in Fig. 1g, the ^2^H peak of D_2_O initially locates at 4.7062 ppm, while it shifts to 4.7445 ppm upon introducing ZnSO_4_ into D_2_O solvent, indicating a reduction in electronic density of hydrogen due to the charge transfer between Zn^2+^ and H_2_O [47]. Subsequently, with the addition of 20 mmol L^−1^ Phe into the ZSO electrolyte, the peak position recovers to 4.7396 ppm. It gradually shifts to 4.7358 ppm with increased Phe concentration (up to 100 mmol L^−1^), suggesting that the partially confined H_2_O molecules within the Zn^2+^ solvation sheath are liberated. It is ascribed to the strong coordination effect between Phe molecule and Zn^2+^ ions that facilitated by higher binding energy [48]. Furthermore, in the Raman spectra analysis (Fig. 1h), the characteristic peak at 977 cm^−1^ is associated with the SO_4_^2−^ (*v*(SO_4_^2−^)), which can be deconvoluted into solvent-separated ion pair (SSIP) and contact ion pair (CIP) (Fig. S7) [48]. The SSIP/CIP ratio increases gradually with escalating Phe addition, reaching 1.83 at 100 mmol L^−1^ Phe concentration. This outcome confirms the involvement of Phe in the CIP by displacing SO_4_^2−^ [48]. Additionally, the impact of Phe additive on Zn^2+^ coordination structure was corroborated by FTIR analysis (Fig. 1i). The vibration stretching of SO_4_^2−^ (*v*(SO_4_^2−^)) undergoes a noticeable blue shift from 1059.7 cm^−1^ (ZSO (s)) to 1080.2 cm^−1^ (ZSO (aq)) upon dissolving ZnSO_4_ powder in H_2_O solvent. This shift indicates weakened interaction between Zn^2+^ and SO_4_^2−^ induced by primary Zn^2+^ solvation sheath formation [25]. Upon introducing 20 mmol L^−1^ Phe into the ZSO electrolyte, the *v*(SO_4_^2−^) shifts to 1081.9 cm^−1^ and further increases with higher Phe concentrations, reaching 1085.6 cm^−1^ at 100 mmol L^−1^ Phe additives. This indicates that the Phe additive weakens the electrostatic coupling between SO_4_^2−^ and Zn^2+^ [25], confirming its influence on Zn^2+^ coordination structure. Furthermore, in the long-wavelength region of 2800–3700 cm^−1^ assigns three characteristic peaks to the O–H stretching vibration, corresponding to strong H-bond (3147 cm^−1^), weak H-bond (3307 cm^−1^), and non-H-bond (3477 cm^−1^) [30, 48], respectively (Fig. S8). As obtained from the H-bond proportion curves (Fig. S9), the intensity of strong H-bond gradually declines with Phe addition increasing, whereas the proportion of weak H-bond shows an upward trend. These observations suggest a suppression in the reaction activity of H_2_O molecules.

### Characterization of the SEI Chemistry

**Fig. 2:**
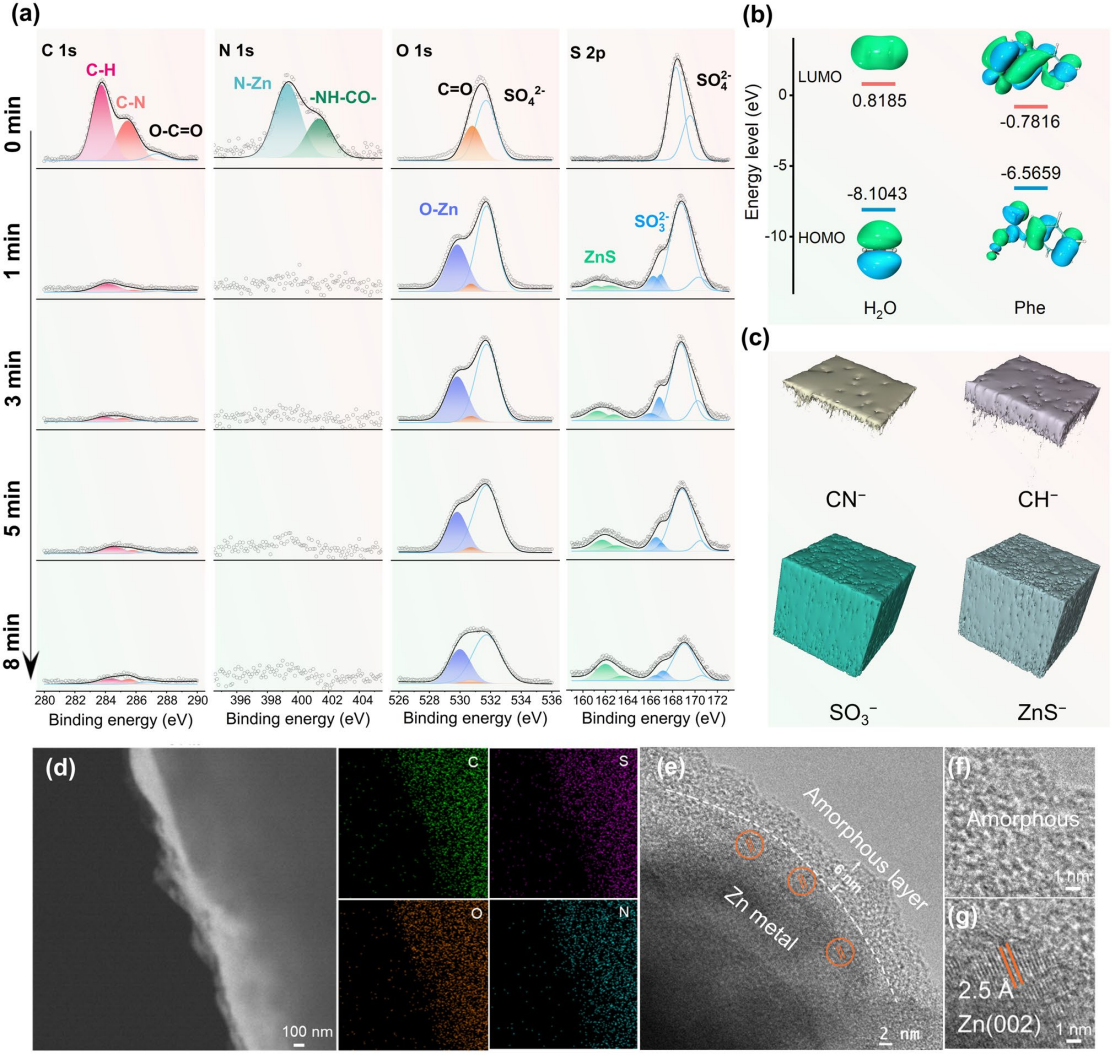
Characterization of SEI chemistry. **a** XPS depth profile of C 1*s*, N 1*s*, O 1*s* and S 2*p* for Zn anode cycled in ZSO/Phe electrolyte for 5 cycles at a current density of 1 mA cm^−1^. **b** HOMO − LUMO energy levels of Phe and water molecules. **c** 3D visualization of TOF–SIMS for CH^−^, CN^−^, SO_3_^−^, and ZnS^−^ in ZSO/Phe electrolyte. **d** HAADF image. **e–g** HRTEM image of the electrode interface and corresponding elemental mapping

To analyze the electrolyte/electrode interface, the TOF–SIMS andXPS with Ar^+^ sputtering depth profiling were employed. As shown in the XPS spectra of N 1*s* (Fig. 2a), the N-Zn (399.3 eV) and –NH-CO– (401.4 eV) bands exclusively exist at the top surface (0 min, before sputtering) of Zn anode cycled in ZSO/Phe electrolyte, demonstrating the chemical adsorption of Phe molecules onto the Zn metal anode surface [35]. It is obviously observed that the ZSO/Phe electrolyte exhibits better wettability at 86.9° than ZSO electrolyte at 96.8°, which further confirms the strong adsorption ability of Phe molecules on Zn anode surface (Fig. S10). However, the contact angle between ZSO/Phe electrolyte and presoaked Zn anode of Phe-contained aqueous solution increases to 98.6°, exhibiting a hydrophobic tendency. As a contrast, the contact angle between ZSO/Phe electrolyte and presoaked Zn anode of deionized water remain nearly constant of 84.9°. This is ascribed to the adsorption of Phe molecules on Zn anode, where the hydrophobic benzene ring ligands toward the electrolyte, forming a molecular hydrophobic layer at the Zn/electrolyte interface, contributing to suppressing water-mediated parasitic side reactions. Additionally, the signals of C-H (283.8 eV) and C-N (285.5 eV) in the C 1*s* spectrum are detected at the top surface [23], diminishing significantly after 1 min of Ar^+^ etching. Meanwhile, the characteristic peak of C = O (530.8 eV) in the O 1*s* spectra displays relatively high intensity before sputtering but gradually fades away after 8 min of etching, indicating an organic layer predominantly covering the electrode top surface [48]. The higher highest occupied molecular orbital (HOMO, − 6.5659 eV) and lower lowest unoccupied molecular orbital (LUMO, − 0.7816) of Phe molecules compared to H_2_O molecules (Fig. 2b) promote preferential electron acquisition by adsorbed Phe. These facilitate Phe reduction on the Zn anode surface ahead of H_2_O to form a hydrophobic organic layer with minute quantities of H_2_ release [48]. In contrast, a series of new characteristic peaks in O 1*s* and S 2*p* spectra corresponding to ZnO (529.8 eV) [49], ZnS (161.1/162.5 eV) [50], and ZnSO_3_ (166.3/166.9 eV) [50] emerge after 1 min of Ar^+^ etching, and the intensities strengthen with increased etching time. This phenomenon demonstrates the *in situ* formation of ZnO, ZnS, and ZnSO_3_ inorganic layers at the bottom of SEI. As shown in Fig. S11, the Phe molecule delivers higher binding energy of − 4.47 eV with SO_4_^2−^ compared to H_2_O, which enables the SO_4_^2−^ ions migrate to Zn anode surface favorably through Phe adsorption layer to be reduced by H_2_ with *in situ* formation of ZnO–ZnS–ZnSO_3_ inorganic layer [48]. Consequently, the Zn 2*p* spectrum exhibits significantly weaker intensity at the top surface (0 min, before sputtering) compared to the sample under Ar^+^ etching (Fig. S12), attributable to both the chemically adsorbed film and the *in situ* organic SEI layer [51]. Furthermore, the existence of SO_4_^2−^ (168.5/169.9 eV) on the Zn anode surface primarily originates from the precipitation of ZnSO_4_ salt [48]. The TOF–SIMS was conducted to investigate the SEI chemistry induced by the Phe additive. Notably, the normalized intensity of organic CN^−^ and CH^−^ species decreases rapidly with increased sputtering depth, while that of ZnS, ZnO, and ZnSO_3_ inorganic compounds increases significantly (Fig. S13). These observations indicate the uniform coverage of an organic layer composed of CN^−^ and CH^−^ species on the dense ZnS, ZnO, and ZnSO_3_ inorganic layer, forming a gradient structure, as confirmed through three-dimensional (3D) visualization (Figs. 2c, S14, and S15). The organic layer enables the electrode/electrolyte interface with considerable deformability to accommodate the volume changes in repeated charging/discharging processes and excellent hydrophobic properties to protect the Zn anode from corrosion caused by active water molecules. Meanwhile, the ZnO/ZnS/ZnSO_3_-rich bottom layer maintains high mechanical rigidity and rapid Zn^2+^ transport capability, enabling uniform Zn^2+^ ions flux for dendrite-free Zn deposition. On the contrary, the Zn anode cycled in ZSO electrolyte presents thimbleful C element and inorganic ZnO/ZnS/ZnSO_3_ species (Fig. S16). Obviously, almost no N element exists on the surface. To accurately determine the composition and structure of SEI formed in ZSO/Phe electrolyte, high-resolution TEM (HRTEM) was performed. The sample, prepared by depositing Zn^2+^ on copper micro-grids within Phe/ZSO electrolyte, revealed an ultrathin amorphous layer on the outer layer of the electrodeposited Zn particles in the high-angle annular dark-field (HAADF) image (Fig. 2d). This amorphous layer is attributed to the *in situ* formed adsorption layer and SEI induced by the Phe additive. The energy-dispersive X-ray spectroscopy (EDS) mapping confirms the uniform distribution of the *in situ* constructed organic and inorganic hybrid SEI layer composed of C, N, O, and S elements on the surface of the Zn anode (Figs. 2d and S17). Furthermore, HRTEM results reveal that the amorphous organic layer has a thickness of about 6 nm, and numerous lattice fringes of Zn (002) are observed inside the SEI layer (Fig. 2e–g). This suggests that the newly plated Zn is deposited through the protective interphase and preferentially grows with a favorable (002) plane during the initial deposition stage. As a result, a dual effect is achieved, simultaneously suppressing the corrosion of water molecules and inhibiting the formation of Zn dendrites.

### Characterization of the Interfacial Interaction

**Fig. 3:**
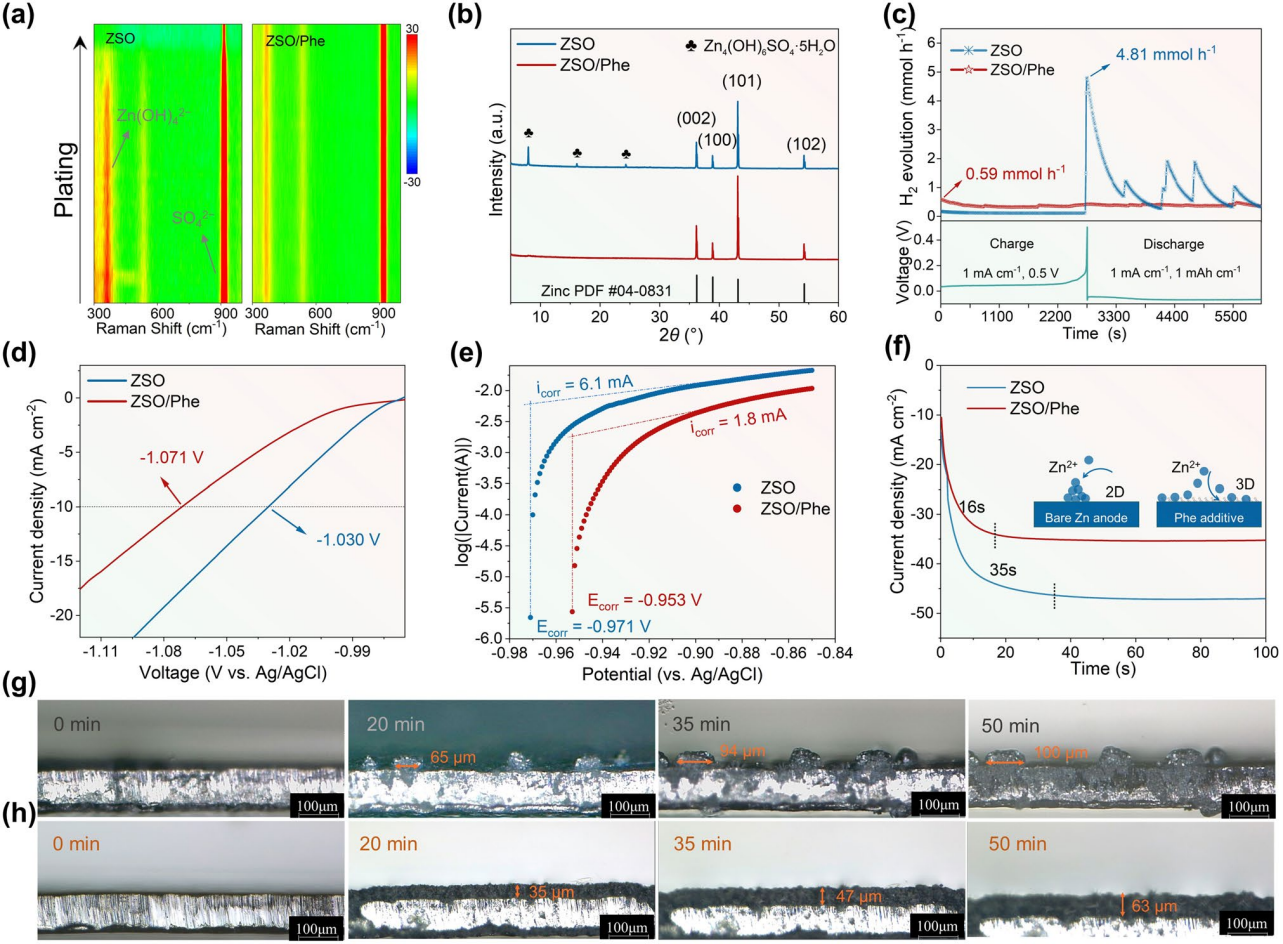
Characterization of interfacial interaction. **a** *In situ* Raman spectrum for Zn anode tested in ZSO and ZSO/Phe electrolytes in Zn^2+^ plating process. **b** XRD patterns of cycled Zn anodes. **c** *In situ* DEMS measurement of H_2_ evolution rate within Zn||Cu cells. **d** LSV curves and **e** Tafel plots of Zn electrodes measured in ZSO and ZSO/Phe electrolytes. **f** CA curves at an overpotential of − 150 mV within Zn||Zn cells (inset: schematic diagrams of 2D and 3D diffusion process of Zn^2+^). *In situ* optical microscopic images of Zn plating process in** g** ZSO and **h** ZSO/Phe electrolytes

To comprehensively comprehend the protective mechanism of the Phe additive in stabilizing the Zn anode and controlling deposition behavior, *in situ* Raman spectroscopy was employed within Zn||Zn symmetrical cells cycled in different electrolytes. As indicated in Fig. 3a, the Zn anode cycled in ZSO electrolyte displays a distinct characteristic peak at 362 cm^−1^, which is attributed to the aggregation of Zn(OH)_4_^2−^ induced by HER [52], revealing the vigorous corrosion reaction during Zn plating. The peak around 916 cm^−1^, attributed to *ν*(SO_4_^2−^) vibration [28], exhibits a slight decrease due to participation in interface side reactions. Conversely, the cell with ZSO/Phe electrolyte did not exhibit Zn(OH)_4_^2−^ by-product enrichment. It is attributed to the collaborative action of the Phe adsorption film, *in situ* SEI formation, and regulation of Zn^2+^ coordination environment that collectively restrains the decomposition of H_2_O molecules. Furthermore, the XRD patterns of Zn anodes cycled in ZSO electrolyte reveal characteristic peaks at 7.9°, 16.1°, and 24.3° corresponding to Zn_4_(OH)_6_ (SO_4_)_4_·5H_2_O by-products, indicating rampant HER at electrode interface (Fig. 3b) [47]. In contrast, a pure Zn anode can be attained in the ZSO/Phe electrolyte, aligning with the *in situ* Raman spectra results. To quantitatively analyze the HER reaction rate, *in situ* differential electrochemical mass spectrometry (DEMS) was carried out on Zn||Cu half cells with different electrolytes. As displayed in Fig. 3c, the H_2_ evolution rate in the ZSO electrolyte sharply increases to 4.81 mmol h^−1^ at 0.5 V, indicating severe corrosion reactions during Zn plating. By contrast, the cell employing ZSO/Phe electrolyte displays significantly suppressed HER, registering only 0.59 mmol h^−1^ at the early charging step. On the one hand, the amino and carboxyl groups are liable to chelate with Zn^2+^ for weakening the reactivity of solvated H_2_O molecules. The lower onset HER potential of ZSO/Phe electrolyte, deduced from linear scanning voltammetry (LSV), further supports the aforementioned results (Fig. 3d). On the other hand, the hydrophobic groups, including benzene ring, and the Phe-induced SEI together constitute a drainage layer that restrains the H_2_O-mediated parasitic side reactions. For an accurate assessment of anti-corrosion capability, corrosion current density and corrosion potential were investigated through Tafel plots (Fig. 3e). Remarkably, the Zn anode in ZSO/Phe electrolyte exhibits a more positive corrosion potential (− 0.953 vs. − 0.971 V) and lower corrosion current density (1.8 vs. 6.1 mA cm^−2^) compared to that in ZSO electrolyte, confirming the superior performance of Phe additive in inhibiting side reactions. Besides, the Zn^2+^ diffusion behavior on the Zn anode surface was analyzed via the chronoamperometry (CA) curves. The current density for Zn||Zn cells tested in ZSO/Phe electrolyte rapidly stabilizes at − 35.4 mA cm^−2^ after a short 2D diffusion process at an overpotential of − 150 mV on the Zn anode (Fig. 3f), demonstrating a sustained 3D diffusion process after nucleation. The hydrophilic units reduce the interfacial free energy between the Zn electrode and the electrolyte while attracting the Zn^2+^ ions migration to Zn anode surface, guiding the Zn^2+^ homogeneous deposition (inset in Fig. 3f). In contrast, the Zn||Zn cell in ZSO electrolyte displays continuous current density increase within 35 s, indicating a rampant 2D diffusion with lateral Zn^2+^ diffusion along the surface and deposition at the most favorable energy position. This may lead to potential safety hazard with dendrite growth and short circuit [49]. Subsequently, the actual Zn^2+^ deposition process was examined by *in situ* optical microscopy, revealing randomly distributed protrusions on the Zn anode surface during electrodeposition in ZSO electrolyte (Fig. 3g). The size of protrusions on the Zn anode surface increases from 65 to 100 μm as deposition time increasing from 20 to 50 min. In addition, distinctly irregular interface is observed on the Zn anode surface after 50 min of plating in the ZSO electrolyte (Fig. S18), resulting in Zn dendrite formation finally. In sharp contrast, a uniform and dense dendrite-free Zn deposition layer with 63 μm thickness is observed on the Zn anode surface after 50 min of plating in the ZSO/Phe electrolyte (Fig. 3h), which further confirms the positive effect of adsorbed Phe molecules and the well-defined SEI layer in promoting uniform Zn^2+^ ions deposition.

### Reversible Zn Plating/Stripping Stability Characterization

**Fig. 4:**
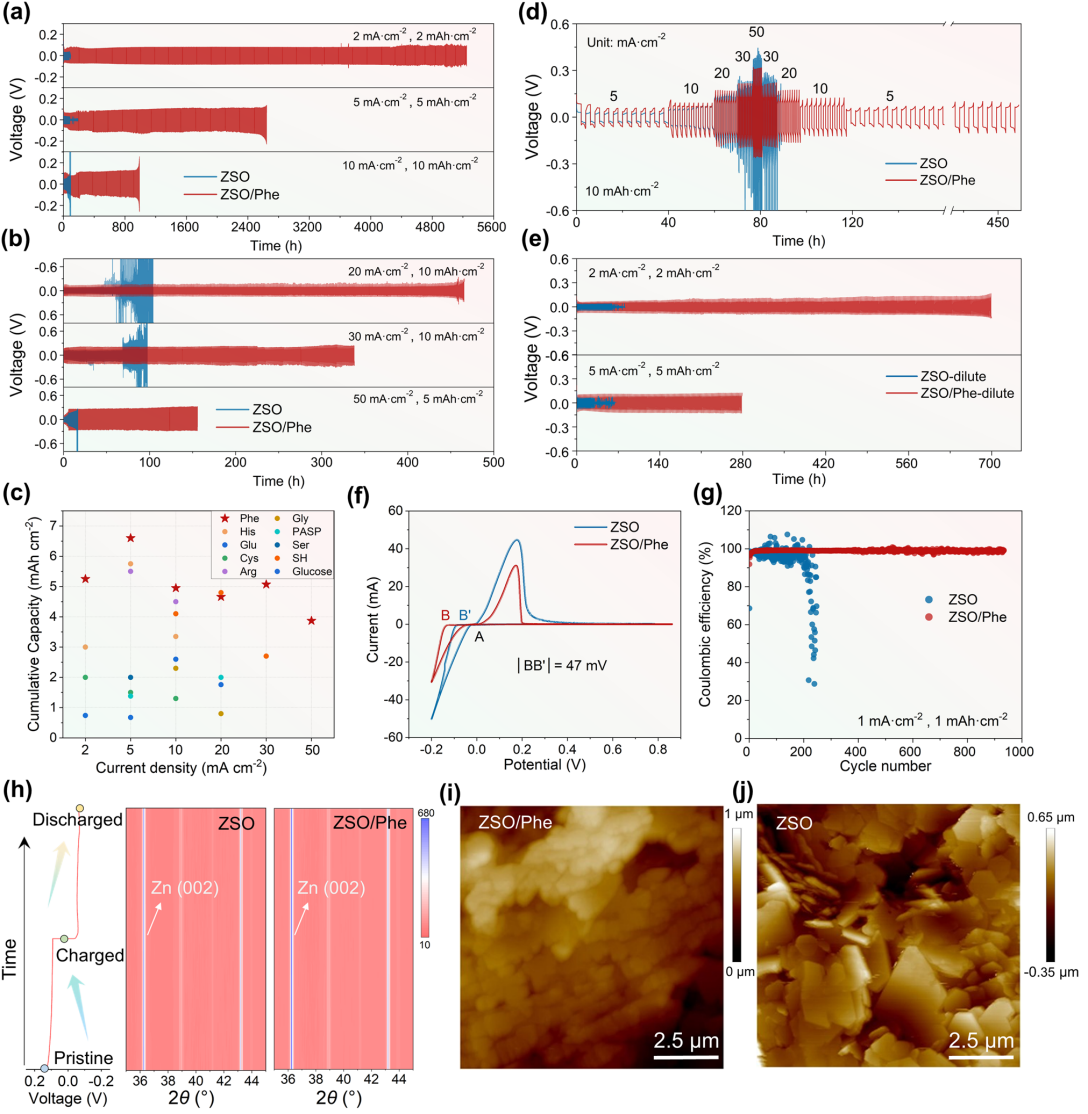
Reversible Zn plating/stripping stability characterization. **a, b** Long-term galvanostatic cycling of Zn||Zn cells at current density of 2, 5, 10, 20, 30, and 50 mA cm^−2^, respectively. **c** Comparison of cumulative plated capacity with previously reported. **d** Rate performances of Zn||Zn cells in different electrolytes. **e** Galvanostatic cycling of Zn||Zn cells in diluted electrolyte. **f** CV curves for Zn nucleation of Zn-Ti cells in different electrolytes. **g** CE of the Zn plating/stripping in Zn||Cu cells. **h** *In situ* XRD measurements on Zn||Zn cells during charging/discharging process. **i, j** AFM images of the cycled Zn in different electrolytes

To certificate the protective effect of Phe additive on the stability and reversibility of Zn anode, extensive galvanostatic cycling experiments were conducted on Zn||Zn symmetrical cells and Zn||Cu half cells. As shown in Fig. 4a, the symmetrical cells with ZSO electrolyte experience rapid short-circuiting after only 80 h of cycling due to rampant dendrite growth (Fig. S19). In contrast, the cells utilizing ZSO/Phe electrolyte demonstrate an extended lifespan exceeding 5250 h with a high overpotential of approximately 138 mV at 2 mA cm^−2^, 2 mAh cm^−2^ (Fig. S20), which is equivalent to an ultrahigh cumulative plated capacity of 5.25 Ah cm^−2^, almost 65 times greater than that achieves using ZSO electrolyte. Optimal protection is achieved with a Phe additive concentration of 20 mmol L^−1^, outperforming concentrations of 50 and 100 mmol L^−1^ (Fig. S21). As indicated in Fig. S22, the Zn||Zn symmetric cells employing 20 mmol L^−1^ Phe electrolyte exhibit greater charge transfer resistance (*R*_ct_) compared with ZSO electrolyte before cycles. This may be attributed to the Phe molecules adsorption layer on Zn anode surface. Significantly, the *R*_ct_ of battery using ZSO/Phe electrolyte decreases after 10 cycles, which implies the enhanced Zn^2+^ conductivity of organic–inorganic dual-protective SEI. On the contrary, the *R*_ct_ of batteries using ZSO electrolyte remarkably increases due to rampant Zn dendrites growth and Zn_4_(OH)_6_ (SO_4_)_4_·5H_2_O insulating species formation that blocks Zn^2+^ transport path. In addition, the EIS curves of Zn||Zn symmetric cells cycled in 20 mmol L^−1^ Phe electrolyte exhibit a smaller *R*_ct_ than 50 and 100 mmol L^−1^ Phe that contributes to improve the electrode reaction kinetics for Zn/Zn^2+^ redox (Fig. S23). Moreover, the protection effect of the Phe additive is validated under more harsh conditions, showcasing superior cycling stability of 2645 h at 5 mA cm^−2^, 990 h at 10 mA cm^−2^, 466 h at 20 mA cm^−2^, 338 h at 30 mA cm^−2^, and 155 h at 50 mA cm^−2^, respectively, (Figs. 4a, b and S24). These cumulative plated capacities surpass most reported Zn metal anode protection strategies, including seven other amino acid additives (Figs. 4c and Table S1) [25, 33, 48, 53–58]. Besides, the rate performances of Zn||Zn symmetrical cells were investigated simultaneously. While the cell with bare electrolyte experiences severe voltage fluctuation at 20 mA cm^−2^, the cell with ZSO/Phe electrolyte maintains a stable voltage profile across various current densities of 5, 10, 20, 30, and 50 mA cm^−2^, sustaining stability for over 460 h even when the current density reverted to 5 mA cm^−2^ (Fig. 4d). This exemplifies the Phe additive's exceptional capability in regulating Zn plating and stripping kinetics. Significantly, even when the electrolyte concentration is diluted tenfold, the Zn anode cycled in ZSO-/Phe-dilute electrolyte also shows stable Zn deposition, surpassing 700 h at 2 mA cm^−2^, 2 mAh cm^−2^, and 279 h at 5 mA cm^−2^, 5 mAh cm^−2^ (Fig. 4e). This reaffirms the practical feasibility of Phe additive utilization in AZIBs. To investigate the nucleation process, cyclic voltammetry (CV) curves were conducted on Zn||Ti half cells. As obtained from Fig. 4f, the cell with ZSO/Phe electrolyte demonstrates an improved nucleation overpotential of 47 mV due to the enhanced reaction energy barrier of Zn deposition caused by the presence of Phe molecules adsorption layer (Fig. S25), which is conducive to drive smaller nucleus radius as well as promote smooth and fine-grained Zn deposits [59]. Consequently, the Zn||Cu half cells maintain excellent Zn plating/stripping reversibility for 934 cycles with an average coulombic efficiency (CE) of 99% (Fig. 4g). The corresponding voltage profiles are displayed in Fig. S26, where moderate voltage hysteresis of 118 mV and stable CE exportation are observed. Contrarily, the cells using ZSO electrolyte experience fluctuating CE, dropping sharply to 30.7% at 218 cycles due to continuous side reactions at deteriorating interfaces. Remarkably, the Zn||Cu half cells cycled in ZSO-/Phe-dilute electrolyte maintain a higher average CE of 98.4% within 218 cycles (Fig. S27). This underscores the overwhelming superiority of the Phe additive in enhancing the reversibility of Zn anode.

Subsequently, *in situ* XRD measurements were taken on Zn||Zn symmetrical cells to explore the Zn deposition behavior optimized by Phe additive. As depicted in Fig. 4h, the Zn anode cycled in ZSO/Phe electrolyte always delivers a stronger characteristic peak of Zn (002) crystal plane at around 36.2° compared to that in ZSO electrolyte during the plating/stripping process, indicating that the Phe molecules adsorption layer guides the preferential Zn nucleation with favorable (002) plane at initial plating stage, thereby inducing Zn^2+^ uniform deposition [47]. This result is consistent with the HRTEM measurement. In addition, the Zn deposition morphology was further investigated by atomic force microscope (AFM). The Zn anode cycled in ZSO electrolyte exhibits numerous lamellar Zn deposition structure with anisotropic (Fig. 4j), which may enlarge the contact areas between electrode and electrolyte, thus leading to severe corrosion reactions without interrupting [60]. Notably, a dense and smooth Zn deposition layer can be observed in ZSO/Phe electrolyte (Fig. 4i), which is responsible for the superior cycling stability and reaction reversibility of Zn anode even in harsh conditions. On larger scales, the SEM measurements of the deposited Zn metal for 5 cycles at 1, 2, and 5 mA cm^−2^ and 5 mA cm^−2^ were taken (Figs. S28 and S29). The Zn anode cycled in ZSO electrolyte exhibits numerous irregular structures, and the deposition morphology tends to get more looser with higher current densities. On the contrary, the Zn anode cycled in ZSO/Phe electrolyte exhibits a smooth and dendrite-free Zn deposition morphology even at higher current density, which further testify the unique regulation of amphipathic groups in Phe compound for dendrite-free Zn deposition.

### Zn||LMO Full Cell Performance

**Fig. 5:**
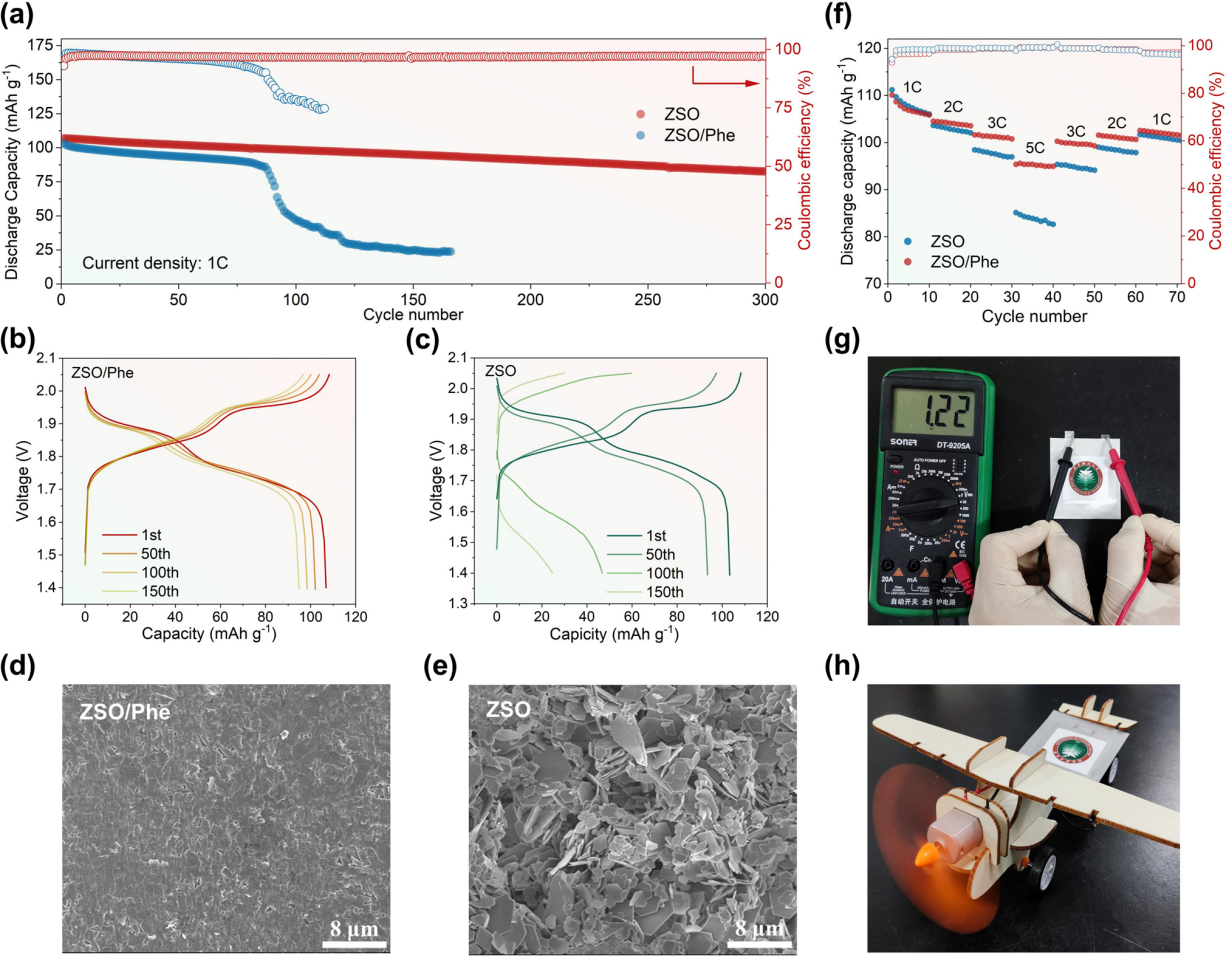
Zn||LMO full cell performance. **a** Long‑term cycling performance at a current density of 1 C. The corresponding voltage–capacity profile in **b** ZSO/Phe and **c** ZSO electrolytes. SEM images of cycled Zn anode in ZSO/Phe **d** ZSO **e** electrolytes. **f** Rate performance at rates of 1, 2, 3, and 5 C. **g, h** Digital photo of open-circuit voltage for the pouch Zn||LMO cell and a model airplane in action powered by Zn||LMO cell

Ultimately, the electrochemical performances of the full cells comprising LMO cathode and Zn anode were tested to validate the practicability of Phe-contained electrolytes. The CV curves of Zn||LMO full cells using ZSO and ZSO/Phe electrolytes all exhibit a pair of redox peaks (Fig. S30). Figure 5a displays the long-term cycling performance of full cells employing ZSO/Phe electrolyte at current density of 1 C (148 mA g^−1^), where a considerable initial capacity of 106.9 mAh g^−1^ and a high capacity retention of 77.3% after 300 cycles are achieved, significantly outperforming the ZSO system that retaining only 27.9% of the initial capacity after 125 cycles. Benefiting from the triple protection of Phe additive, including solvation structure regulation, amphipathic molecular adsorption, and organic–inorganic hybrid SEI formation, the Zn||LMO full cell delivers highly consistent charge/discharge profile (Fig. 5b) and smooth Zn anode (Fig. 5d). In sharp contrast, the severe dendrite growth and corrosion sites occurred in the ZSO system (Fig. 5e) may gobble up the Zn anode while exhausting the electrolyte, undoubtedly resulting in the capacity fading and CE decreasing (Fig. 5c). Furthermore, the ZnSO_4_-based electrolyte concentration is diluted by 10 times to testify the modification of Phe additive on reversibility of Zn anode, where the Zn||LMO full cells employing ZSO-/Phe-dilute electrolyte exhibits stable capacity output that 55% capacity retention after 100 cycles (Fig. S31), whereas the system of ZSO-dilute electrolyte without additive participation displays a sharp capacity decline and lower CE at initial charging/discharging process. It is more notable that the Zn||LMO full cells with ZSO/Phe electrolyte display superior rate performance, corresponding to a higher average discharge capacity of 95.2 mAh g^−1^ with CE of 99.2% at 5 C and a reversible specific capacity of 102.1 mAh g^−1^ after returning to 1 C (Fig. 5f), which outperforms the ZSO system of exhibiting lower rate capacity of 83.8 mAh g^−1^ at 5 C. Subsequently, a single-layer pouch cell was assembled with ZSO/Phe electrolyte to successfully drive the airplane model moving toward (Fig. 5g, h), which further verify the practical application of Phe additive in AZIBs.

## Conclusion

In conclusion, we have introduced the highly biocompatible amino acids molecules as electrolyte additive into AZIBs to address the issues of Zn anode corrosion and regulate the Zn^2+^ uniform deposition. The Phe molecule featuring benzene ring ligands embraces higher adsorption energy for Zn anode to constitute a protective layer for synergistically homogenizing Zn^2+^ distribution and insulating water molecules, while the adjacent hydroxyl and carboxyl groups incline to form chelating bonds with Zn^2+^ through lone pair electrons, rendering efficient solvation based on Zn^2+^-Phe coordination for weakening the reactivity of H_2_O molecules. Moreover, the preferential reduction of Phe molecules prior to H_2_O promotes *in situ* formation of organic–inorganic hybrid SEI for enhancing the interfacial stability of the Zn anode. Benefiting from the triple protection of Phe containing electrolyte, all of the Zn||Zn, Zn||Cu, and Zn||LMO cells display significantly improved electrochemical performances. Even at extreme diluted electrolytes, the Zn anode still exhibits an ultralong cycle life of 700 h under the condition of 2 mA cm^−2^, 2 mAh cm^−2^, confirming the feasibility of the electrolyte engineering for practical applications in AZIBs.

## Supplementary Information

Below is the link to the electronic supplementary material.

Supplementary file1 (PDF 1554 KB)
