# Supplementary material for: Amphipathic Phenylalanine-Induced Nucleophilic–Hydrophobic Interface Toward Highly Reversible Zn Anode
Source: Nanomicro Lett. 2024 Mar 28;16:164. doi: 10.1007/s40820-024-01380-x (PMC10978566; doi:10.1007/s40820-024-01380-x)
Supplement: Supplementary file 1 — Supplementary file1 (PDF 1554 KB) [file 40820_2024_1380_MOESM1_ESM.pdf]

Supporting Information for

Amphipathic Phenylalanine Induced Nucleophilic-Hydrophobic Interface towards Highly Reversible Zn Anode

Anbin Zhou[1,+], Huirong Wang[1,+], Fengling Zhang[1], Xin Hu[1], Zhihang Song[1], Yi Chen[1], Yongxin Huang[1, 2,*], Yanhua Cui[4], Yixiu Cui[4], Li Li[1, 2, 3], Feng Wu[1, 2, 3], Renjie Chen[1, 2, 3,*]

[1]Beijing Key Laboratory of Environmental Science and Engineering, School of Materials Science & Engineering, Beijing Institute of Technology, Beijing 100081, P. R. China

[2]Advanced Technology Research Institute, Beijing Institute of Technology, Jinan 250300, P. R. China

[3]Collaborative Innovation Center of Electric Vehicles in Beijing, Beijing 100081, P. R. China

[4]Institute of Electronic Engineering, China Academy of Engineering Physics, Mianyang 621900, P. R. China

[+]Anbin Zhou and Huirong Wang contributed equally to this work.

*Corresponding authors. E-mail: huangyx@bit.edu.cn (Yongxin Huang); chenrj@bit.edu.cn (Renjie Chen)

Supplementary Figures

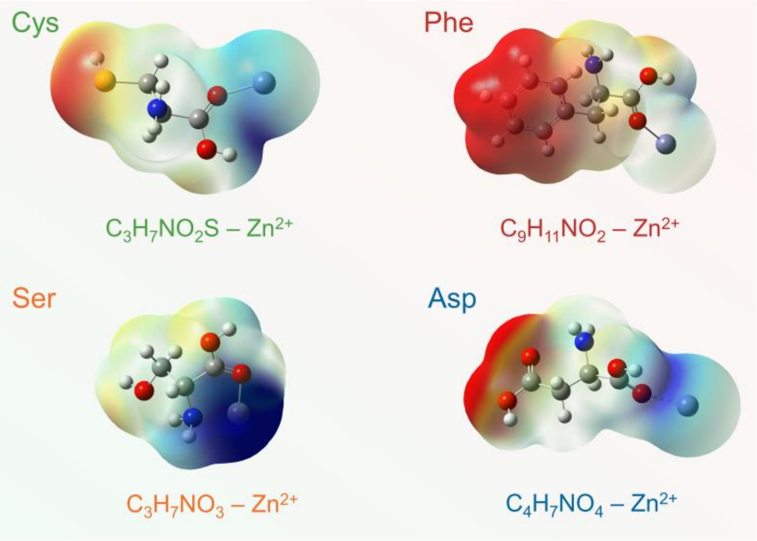

Fig. S1 Electrostatic potential mapping of Phe/Asp/Ser/Cys molecules within interaction with Zn^{2+}

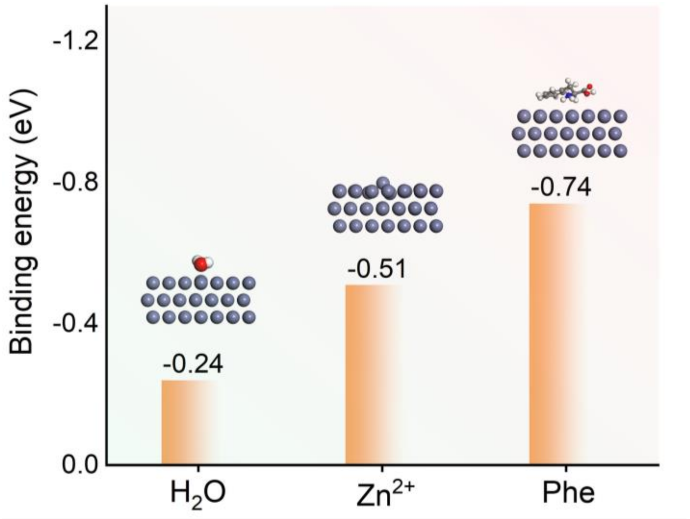

Fig. S2 The adsorption energy of Phe, Zn^{2+}, and H_2O on the Zn (002) facet

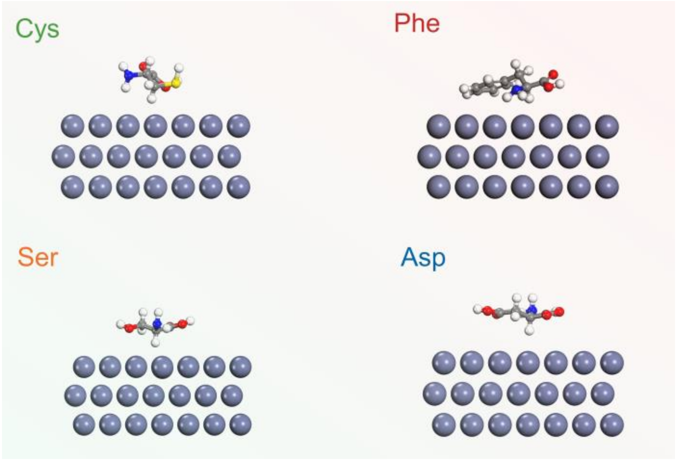

Fig. S3 The adsorption model of Phe/Asp/Ser/Cys molecules on Zn (002) facet

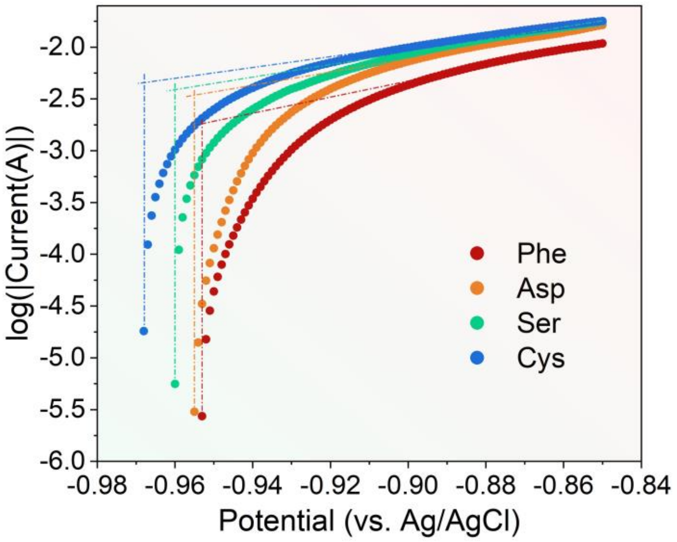

Fig. S4 Tafel plots of the Zn electrodes measured in ZSO electrolytes with 20 mmol L^{-1} Phe/Asp/Ser/Cys additives

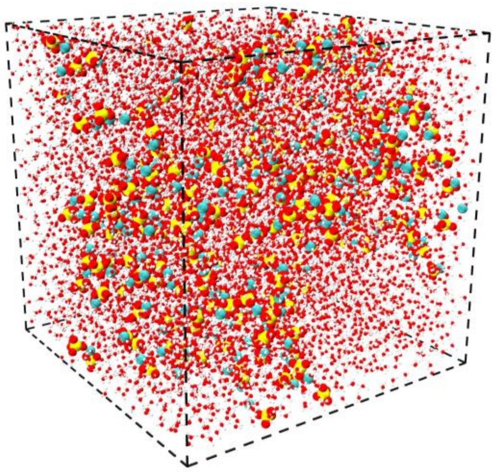

Fig. S5 Images of ZSO system obtained from molecular dynamics simulations

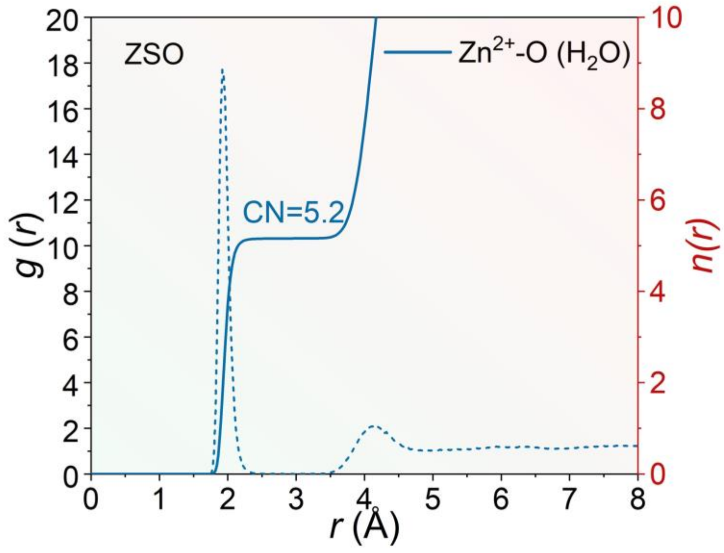

Fig. S6 The radial distribution functions for Zn^{2+}-O (H_2O) in ZSO electrolyte

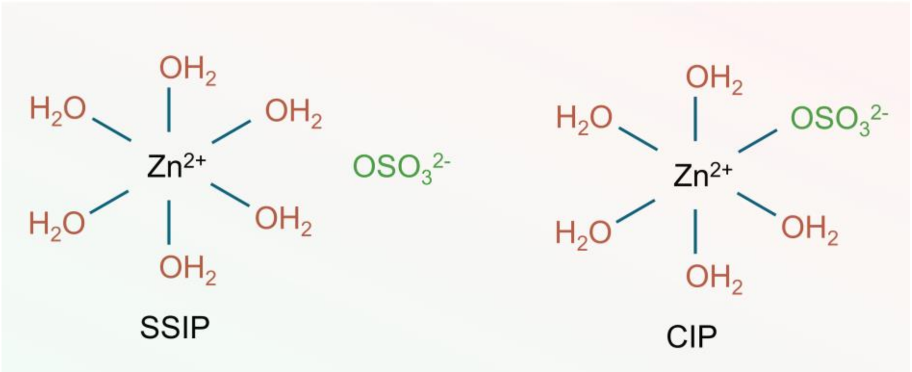

Fig. S7 Schematic illustration of Zn^{2+} solvation structures in ZSO/Phe electrolyte

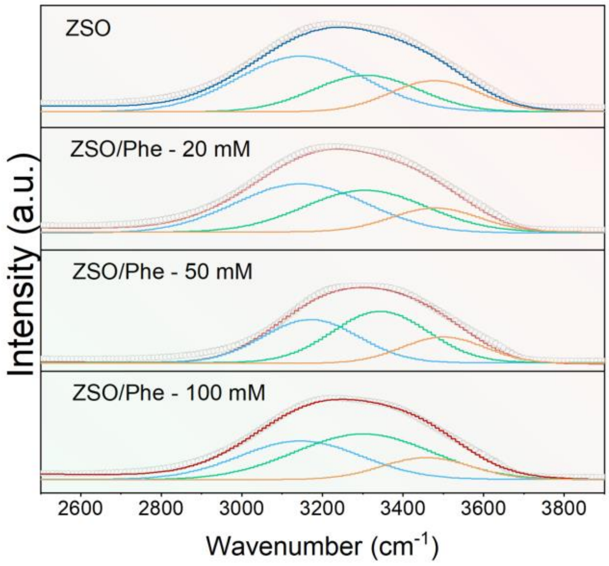

Fig. S8 FTIR spectra for characteristic peaks assigned to the O-H stretching vibration in ZSO and ZSO/Phe system

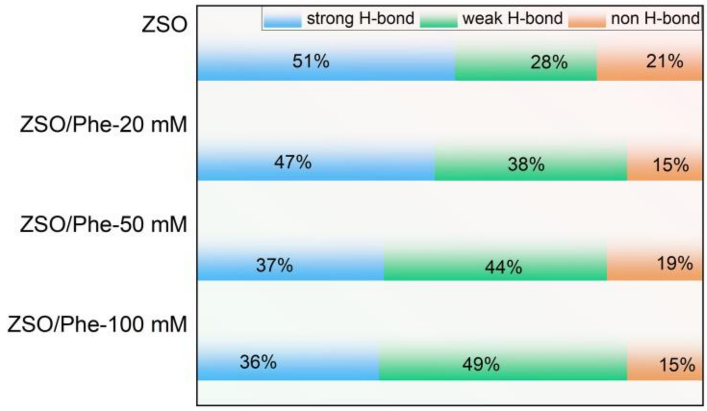

Fig. S9 The H-bond proportion graphs obtained from FTIR spectra

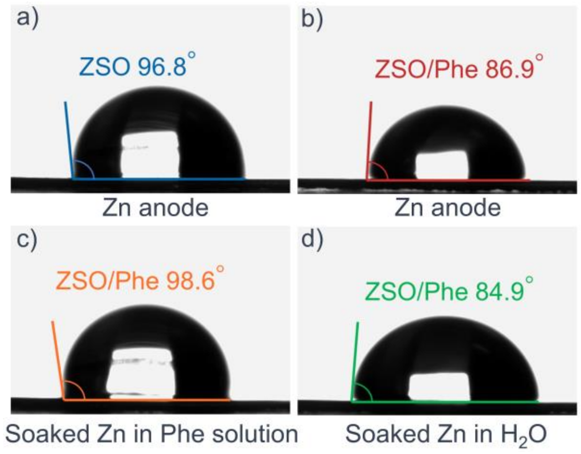

Fig. S10 Contact angles of ZSO electrolyte (**a**) and ZSO/Phe electrolyte (**b**) on Zn anode, and ZSO/Phe electrolyte on pre-soaked Zn anode of Phe-contained aqueous solution (**c**), and ZSO/Phe electrolyte on pre-soaked Zn anode of deionized water (**d**)

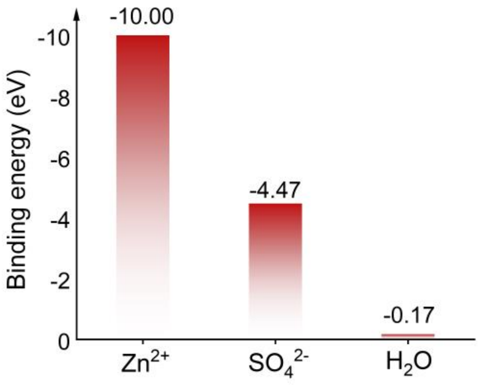

Fig. S11 Binding energy for Phe with SO_4^{2-}, Zn^{2+} and H_2O molecule by the density functional theory calculation

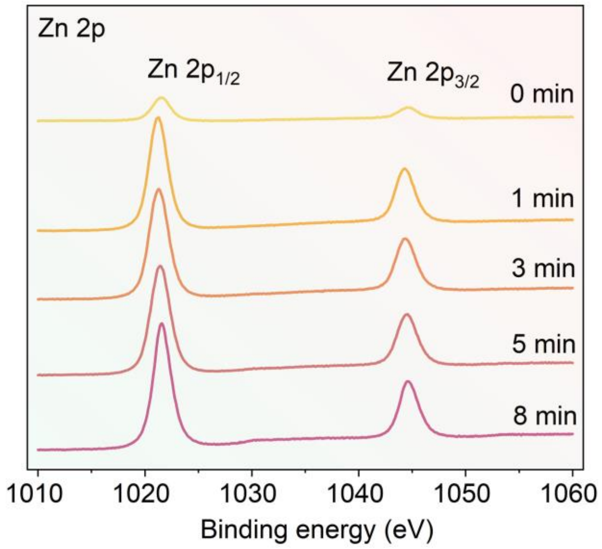

Fig. S12 XPS depth profile of Zn 2p for Zn anode cycled in ZSO/Phe electrolyte

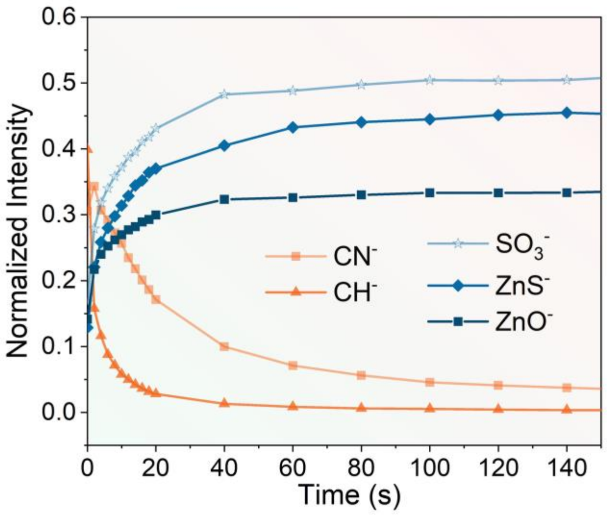

Fig. S13 The normalized intensity of organic CN^- and CH^- and inorganic ZnS^-, ZnO^-, and SO_3^- species

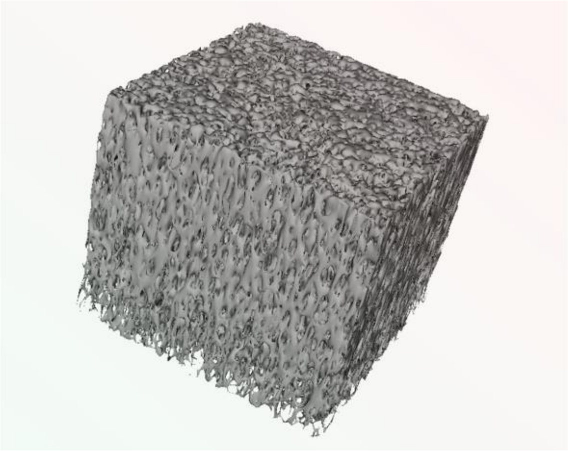

Fig. S14 3D visualization of TOF-SIMS for Zn

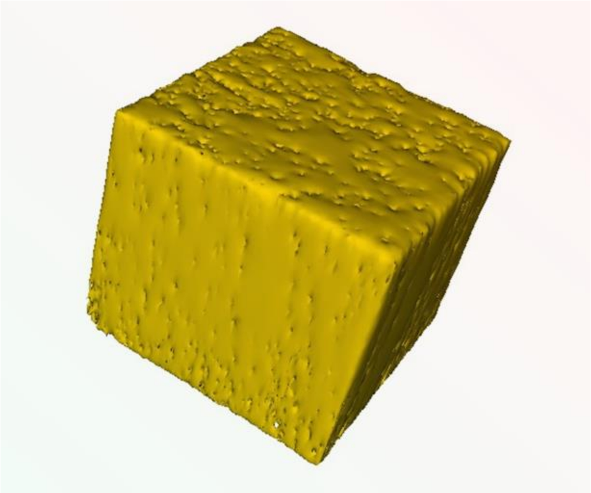

Fig. S15 3D visualization of TOF-SIMS for ZnO^-

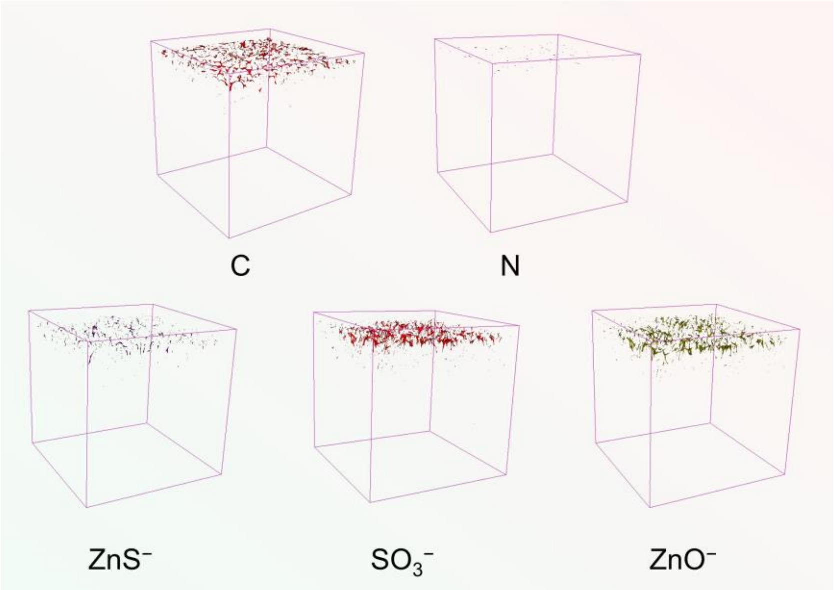

Fig. S16 3D visualization of TOF-SIMS for C, N elements, ZnS^-, SO_3^-, and ZnO^- species in ZSO electrolyte

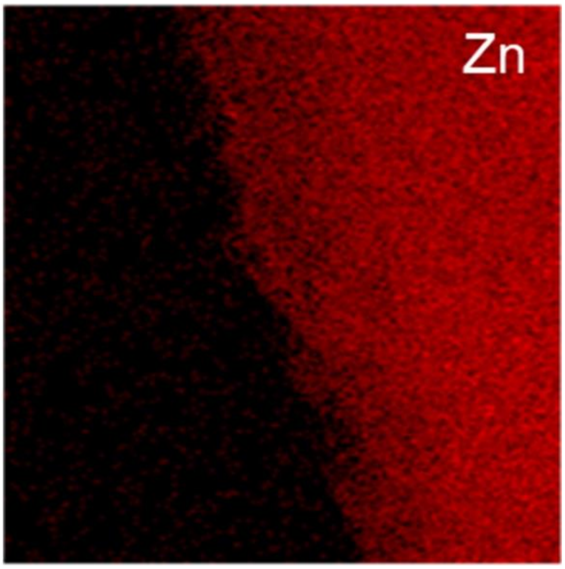

Fig. S17 HRTEM image of the electrode interface and the Zn elemental mapping

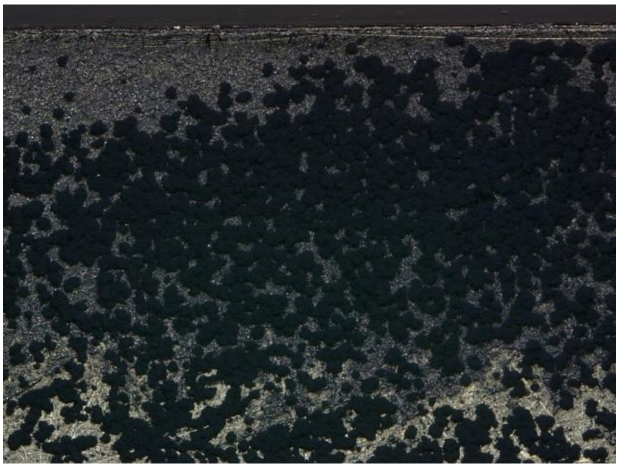

Fig. S18 The deposition morphology of Zn anode electrodeposited in ZSO electrolyte

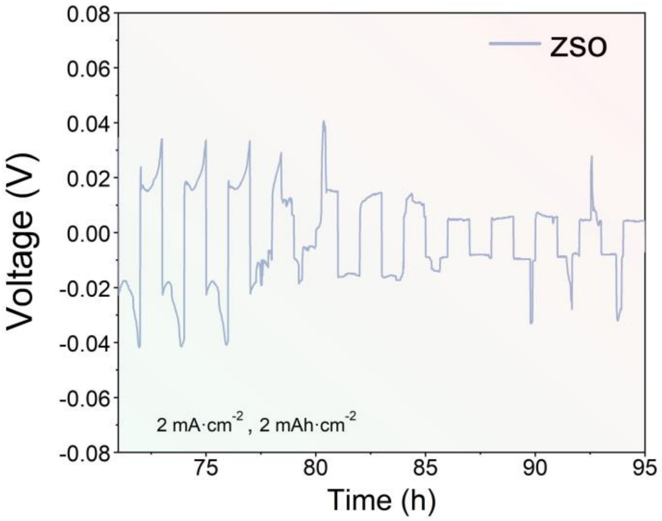

Fig. S19 The galvanostatic cycling of Zn||Zn cells in ZSO electrolyte

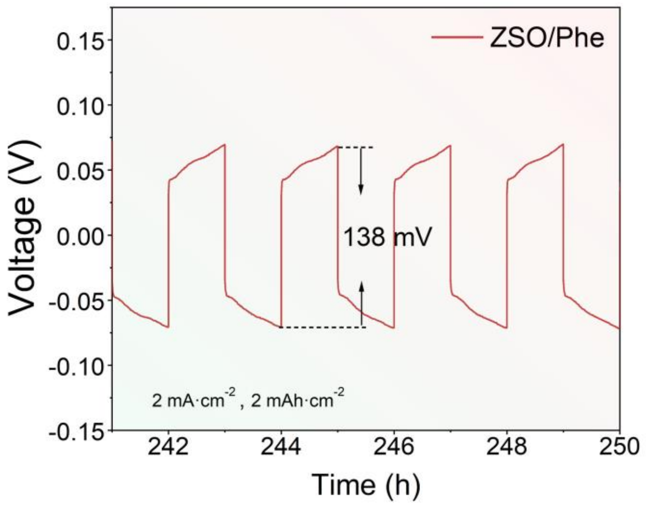

Fig. S20 The galvanostatic cycling of Zn||Zn cells in ZSO/Phe electrolyte

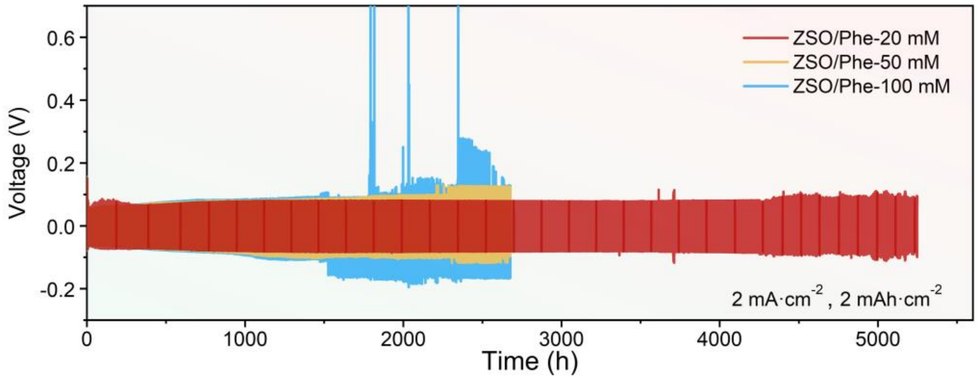

Fig. S21 The galvanostatic cycling of Zn||Zn cells in different Phe concentration electrolytes

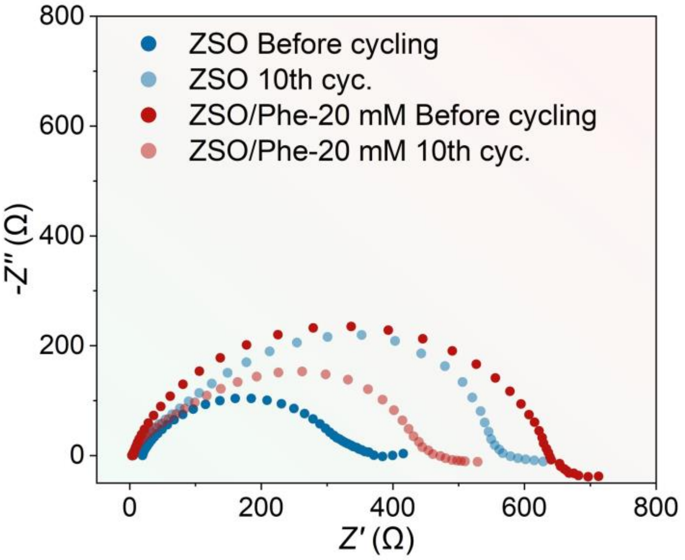

Fig. S22 EIS curves of Zn||Zn symmetric cells using ZSO and ZSO/Phe-20 mM electrolytes before and after 10 cycles

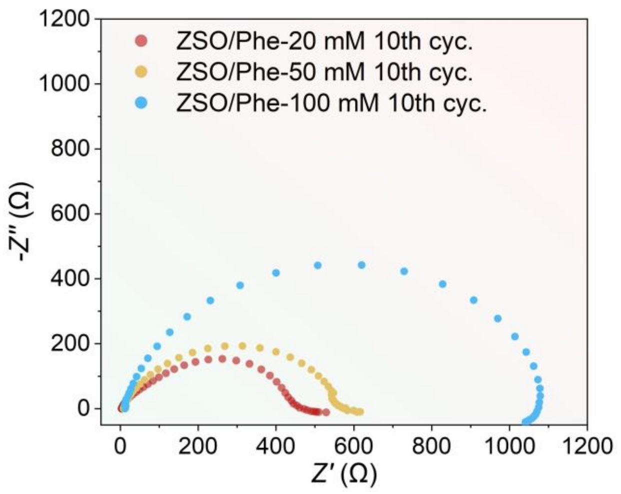

Fig. S23 EIS curves of Zn||Zn symmetric cells using ZSO/Phe-20 mM, ZSO/Phe-50 mM and ZSO/Phe-100 mM electrolytes after 10 cycles

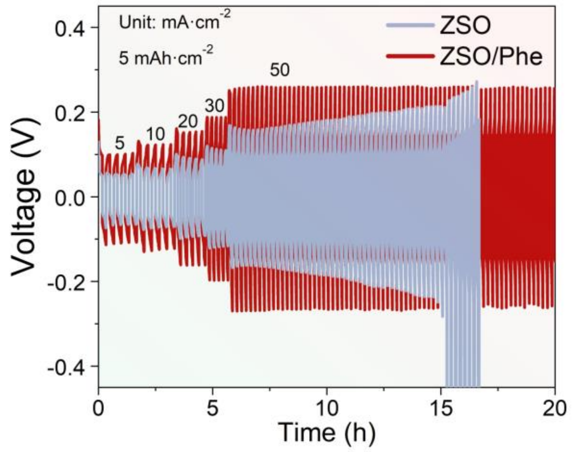

Fig. S24 The galvanostatic cycling of Zn||Zn cells under condition of 50 mA cm^{-2}, 5 mAh cm^{-2} in the initial stage

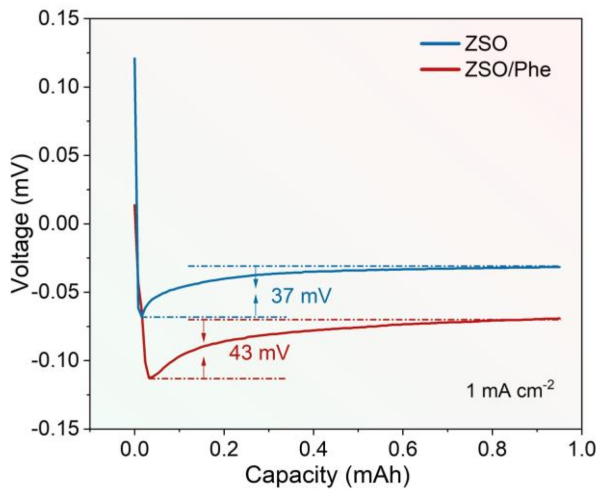

Fig. S25 The nucleation overpotential tested in Zn||Cu half cells

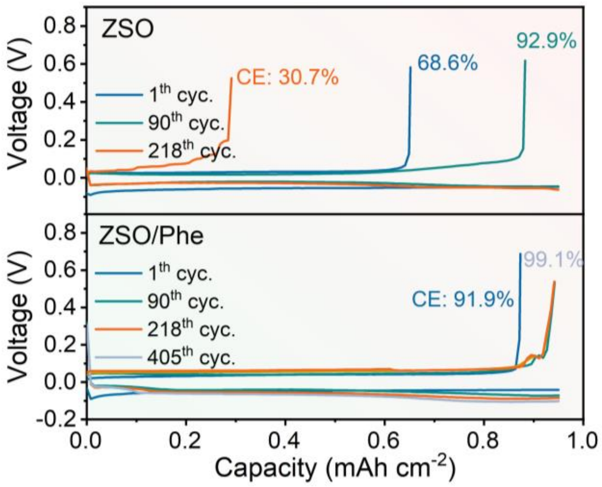

Fig. S26 The corresponding voltage profiles for CE measurement in Zn||Cu cells

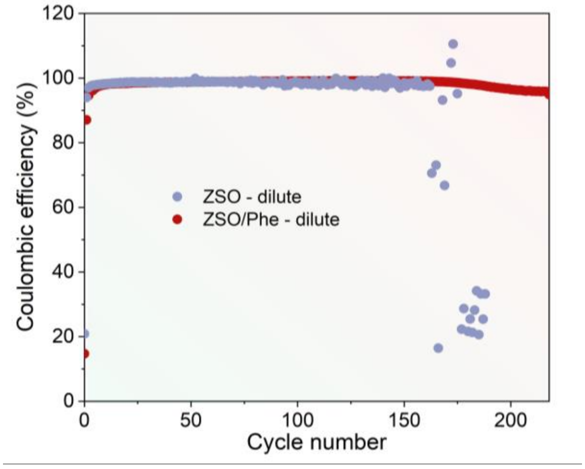

Fig. S27 The CE of the Zn plating/stripping in dilute electrolytes

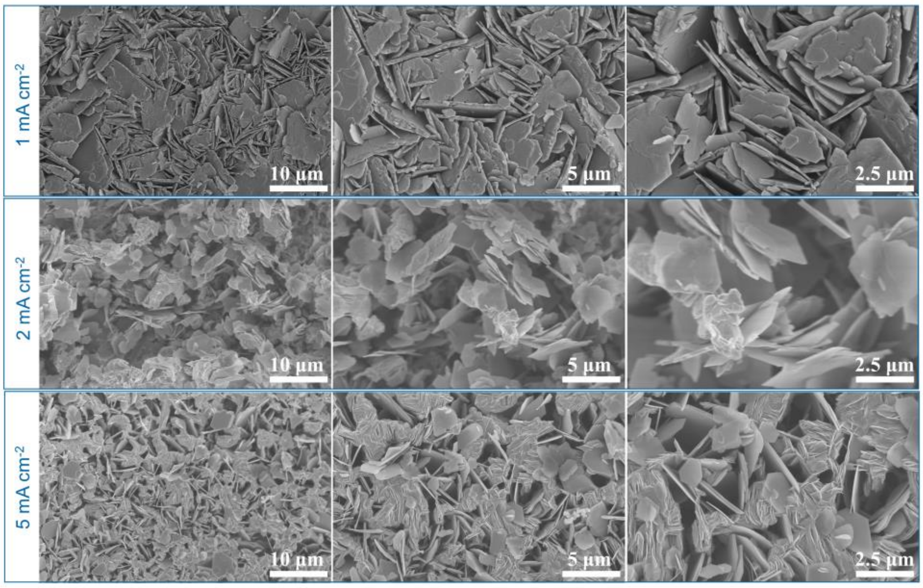

Fig. S28 SEM images of the deposited Zn metal for 5 cycles at 1 mA cm^{-2}, 2 mA cm^{-2} and 5 mA cm^{-2} in ZSO electrolyte

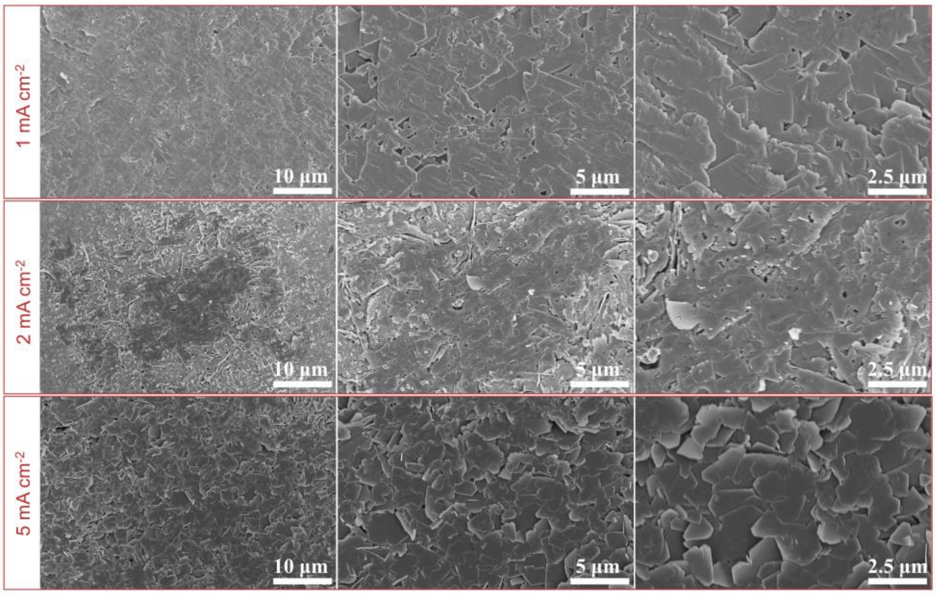

Fig. S29 SEM images of the deposited Zn metal for 5 cycles at 1 mA cm^{-2}, 2 mA cm^{-2} and 5 mA cm^{-2} in ZSO/Phe electrolyte

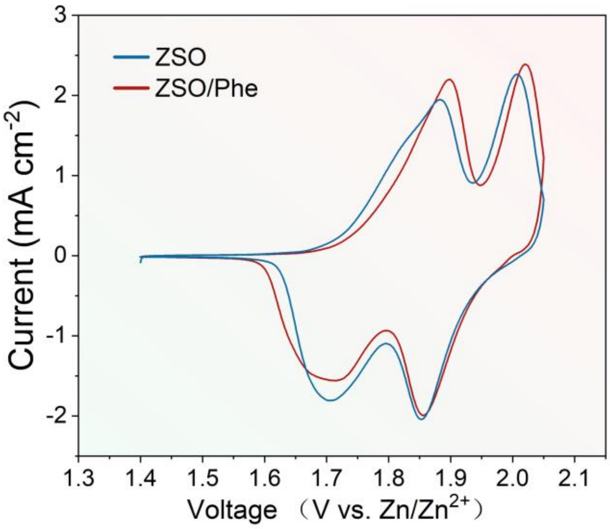

Fig. S30 CV curves of Zn||LMO full cells with ZSO and ZSO/Phe electrolytes

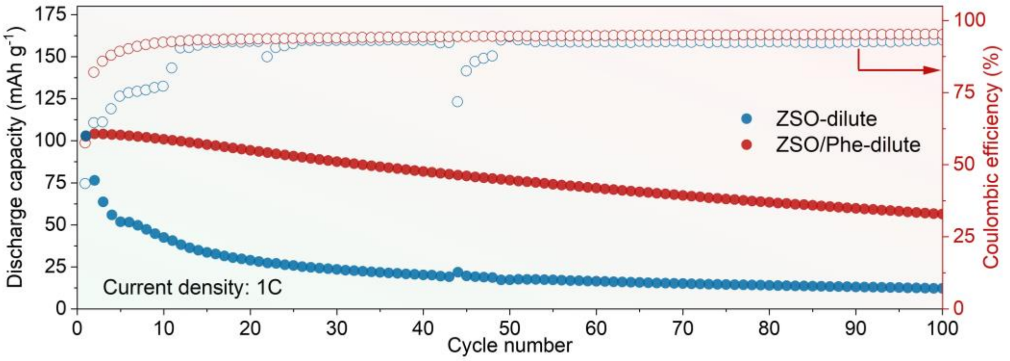

Fig. S31 Zn||LMO full cells cycling performance in dilute electrolytes

Table S1 Comparison of the cycling stability of Zn||Zn cells with previous reports

Additives	Test condition	Lifespan	Cumulative Capacity (Ah cm^{-2})	References
Phe	2 mA cm^{-2}, 2 mAh cm^{-2}	5250 h	5.25 Ah cm^{-2}	This work
	5 mA cm^{-2}, 5 mAh cm^{-2}	2645 h	6.6 Ah cm^{-2}	
	10 mA cm^{-2}, 10 mAh cm^{-2}	990 h	4.95 Ah cm^{-2}	
	20 mA cm^{-2}, 10 mAh cm^{-2}	466 h	4.66 Ah cm^{-2}	
	30 mA cm^{-2}, 10 mAh cm^{-2}	338 h	5.07 Ah cm^{-2}	
	50 mA cm^{-2}, 5 mAh cm^{-2}	155 h	3.87 Ah cm^{-2}	
His	2 mA cm^{-2}, 2 mAh cm^{-2}	3000 h	3 Ah cm^{-2}	[54]
	5 mA cm^{-2}, 2 mAh cm^{-2}	2300 h	5.75 Ah cm^{-2}	
	10 mA cm^{-2}, 4 mAh cm^{-2}	670 h	3.35 Ah cm^{-2}	
Glu	10 mA cm^{-2}, 10 mAh cm^{-2}	520 h	2.6 Ah cm^{-2}	[56]
	20 mA cm^{-2}, 20 mAh cm^{-2}	176 h	1.76 Ah cm^{-2}	
Cys	2 mA cm^{-2}, 1 mAh cm^{-2}	2000 h	2 Ah cm^{-2}	[57]
	5 mA cm^{-2}, 5 mAh cm^{-2}	600 h	1.5 Ah cm^{-2}	
	10 mA cm^{-2}, 10 mAh cm^{-2}	260 h	1.3 Ah cm^{-2}	
Arg	5 mA cm^{-2}, 4 mAh cm^{-2}	2200 h	5.5 Ah cm^{-2}	[33]
	10 mA cm^{-2}, 4 mAh cm^{-2}	900 h	4.5 Ah cm^{-2}	
Gly	10 mA cm^{-2}, 10 mAh cm^{-2}	460 h	2.3 Ah cm^{-2}	[48]
	20 mA cm^{-2}, 20 mAh cm^{-2}	80 h	0.8 Ah cm^{-2}	
PASP	5 mA cm^{-2}, 2.5 mAh cm^{-2}	550 h	1.38 Ah cm^{-2}	[55]
	20 mA cm^{-2}, 1 mAh cm^{-2}	200 h	2 Ah cm^{-2}	
Ser	5 mA cm^{-2}, 5 mAh cm^{-2}	800 h	2 Ah cm^{-2}	[53]
SH	10 mA cm^{-2}, 10 mAh cm^{-2}	820 h	4.1 Ah cm^{-2}	[58]
	20 mA cm^{-2}, 20 mAh cm^{-2}	480 h	4.8 Ah cm^{-2}	
	30 mA cm^{-2}, 10 mAh cm^{-2}	180 h	2.7 Ah cm^{-2}	
Glucose	2 mA cm^{-2}, 2 mAh cm^{-2}	740 h	0.74 Ah cm^{-2}	[25]
	5 mA cm^{-2}, 5 mAh cm^{-2}	270 h	0.675 Ah cm^{-2}	
